# The effect of combined rhythmic breathing and aromatherapy on pain intensity and anxiety in patients with unstable angina

**DOI:** 10.1016/j.conctc.2025.101575

**Published:** 2025-11-25

**Authors:** Sahar Nouri, Nasrin Hanifi, Farhad Ramezani-Badr

**Affiliations:** Critical Care and Emergency Nursing Department, Nursing and Midwifery School, Zanjan University of Medical Sciences, Zanjan, Iran

**Keywords:** Breathing exercises, Aromatherapy, Pain, Anxiety, Unstable angina

## Abstract

**Background:**

Unstable angina is a serious heart condition characterized by intense chest pain and anxiety, which substantially reduces patients’ well-being.

**Objective:**

This study examined the combined effects of rhythmic breathing and aromatherapy on pain and anxiety in unstable angina.

**Methods:**

This two-arm randomized controlled trial involved 56 participants, who were equally allocated to an intervention group (n = 28) and a control group (n = 28). The intervention group received rhythmic breathing exercises combined with aromatherapy using 40 % diluted damask rose essential oil, administered in three sessions per hour over a 3-h period. **Pain and anxiety were measured using Visual Analog Scales at baseline and at 1-, 2-, and 3-h post-intervention.**

**Results:**

Repeated measures ANOVA indicated a significant time-based decrease in pain and anxiety within the intervention group, contrasting with the control group (P < .001).

**Conclusion:**

Pain and anxiety were effectively reduced in unstable angina patients through a combined intervention of rhythmic breathing and aromatherapy. These results highlight the promise of non-drug methods for treating the mental and physical effects of heart disease.

## 1 Introduction

Cardiovascular diseases (CVDs) represent a leading cause of global mortality, accounting for an estimated 20.5 million deaths in 2021. The burden of CVD continues to rise, particularly in low- and middle-income countries, claiming a substantial number of lives annually [1,2]. Iran's Ministry of Health reports that CVDs account for over 40 % of all deaths, with myocardial infarction responsible for 19 % of those [3].

Unstable angina, a frequent sign of heart disease, often presents with intense chest pain and anxiety [4]. These symptoms can severely affect patients' physical and mental well-being [5]. Anxiety's psychological impact is compounded by its effect on the sympathetic nervous system, potentially increasing blood pressure, heart rate, and the risk of a heart attack [6]. Analgesics and anti-anxiety medications are commonly used to manage these symptoms. However, these medications, particularly benzodiazepines used for anxiety relief, are associated with notable adverse effects; for instance, their administration during acute coronary syndromes has been linked to a significantly increased risk of developing posttraumatic stress disorder symptoms, thereby potentially worsening the patient's long-term psychological prognosis [7]. Researchers are increasingly interested in complementary, non-pharmacological techniques as safer, cheaper alternatives in this area. [8,9]. Two notable methods in this respect are rhythmic breathing and aromatherapy [10,11].

Regulating breathing via rhythmic breathing, a basic relaxation method, lessens anxiety and pain by activating the parasympathetic nervous system [12]. This approach has a positive effect on how patients cognitively respond to stress, improving both their psychological and physiological well-being [13]. Research shows rhythmic breathing lowers blood pressure and improves hemodynamic measures [14,15]. Plant-based essential oils, for example, rose oil, are used in aromatherapy, another effective complementary method, to create calming effects [16,17]. Rose oil, containing citronellol and 2-phenylethanol, may relieve anxiety and pain by modulating neurotransmitters such as serotonin and endorphins in the limbic system [18]. This method is prevalent globally, with significant use in holistic nursing [19]. The combination of aromatherapy with rhythmic breathing may offer a holistic approach to easing pain and anxiety in cardiac patients. Aromatherapy complements rhythmic breathing's stress-reducing effects by stimulating the nervous system to trigger a calming chemical response, thereby enhancing its effects on physiological responses. However, the effects of using these two techniques together on unstable angina patients haven't been thoroughly investigated. Studies have shown that each technique individually helps reduce pain and anxiety [19,20]. The purpose of this study was to determine the effects of combining rhythmic breathing and aromatherapy to reduce pain and anxiety in unstable angina patients. These non-pharmacological methods offer new ways to manage patient symptoms and improve nursing care.

## 2 Methodology

### 2.1 Study design and setting

A randomized controlled trial (RCT) with a two-arm design was performed to assess the combined impact of rhythmic breathing and aromatherapy on pain intensity and anxiety in patients with unstable angina. This study was performed in the coronary care units (CCUs) of Ayatollah Mousavi and Imam Hossein Hospitals, Zanjan, Iran, from June to October 2024.

### 2.2 Sample size and recruitment

The sample size was calculated using G∗Power software. With a power of 95 %, an alpha level of .05, and a large effect size of 1 (based on a previous study by Khoshkhati et al.), a minimum of 23 participants per group was required [21]. To account for potential dropouts, 28 participants were recruited for each group.

### 2.3 Participants and eligibility criteria

This study included adult patients aged 30–70 years who were admitted to the coronary care units (CCUs) of Ayatollah Mousavi and Imam Hossein Hospitals (Zanjan, Iran) with a confirmed diagnosis of unstable angina by a cardiologist. Eligible patients were required to have a pain score greater than 3 on the Visual Analog Scale for Pain (VAS-P) and an anxiety score greater than 30 on the Visual Analog Scale for Anxiety (VAS-A).

Exclusion criteria were applied to ensure participant safety and minimize confounding factors. Patients were excluded if they had.

✓ A prior history of coronary angiography or percutaneous coronary intervention
✓ Current dependence on narcotics or psychoactive substances
✓ Known or previously diagnosed psychiatric disorders that could affect psychological assessment
✓ Cognitive impairments or the inability to understand and perform breathing techniques
✓ Respiratory diseases that interfere with deep or rhythmic breathing
✓ Known allergy or sensitivity to rose essential oil

Participants who met the eligibility criteria and agreed to participate signed written informed consent forms before enrollment.

### 2.4 Sampling and randomization

**Fig. 1:**
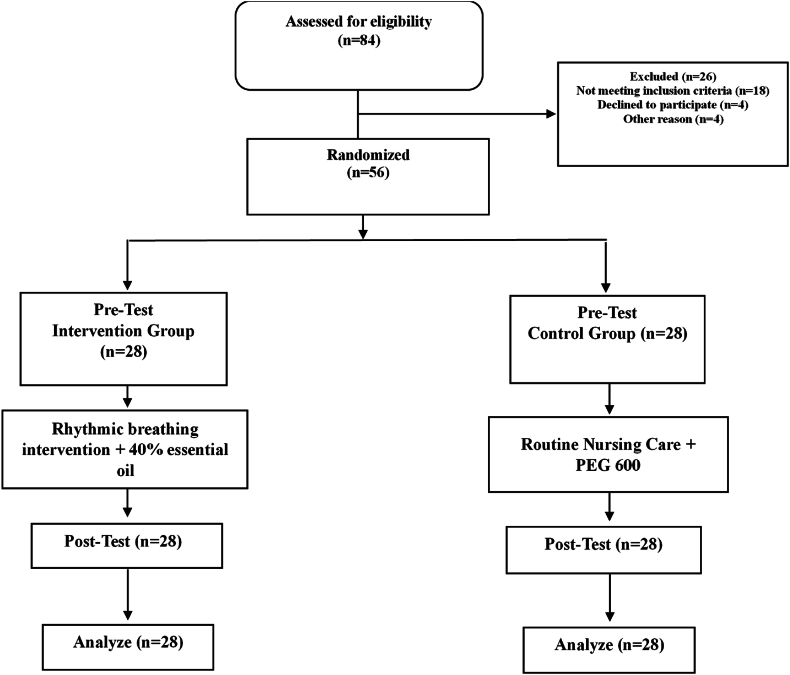
The process of the study according to the CONSORT, LSD flow diagram (2010).

The researchers used convenience sampling to select participants. Consecutively enrolled patients meeting the inclusion criteria were informed of the study's purpose and procedures. All willing participants provided written informed consent. Participants were then randomly assigned to either the intervention or control group using permuted blocks of four. A team member (initials anonymized) created the random allocation sequence online via a secure website (https://www.sealedenvelope.com). Sealed envelopes ensured allocation concealment. Randomly assigned labels, “A” (intervention) or “B” (control), were placed in each envelope. **A research assistant who was not involved in recruitment sequentially opened the envelopes.** Participants were grouped according to the card drawn from their assigned envelope (Fig. 1).

### 2.5 Data collection

A trained research assistant, unaware of group assignments, collected the data. Demographic data, pain, and anxiety were assessed via a questionnaire and the VAS-P and VAS-A scales. Baseline and post-intervention assessments (1, 2, and 3 h) were performed.

### 2.6 Demographic data questionnaire

Demographic data, including age, gender, education, marital status, medical conditions, and analgesic use, were obtained via a structured questionnaire.

### 2.7 Visual Analog Scale for Pain

Pain intensity was self-reported by participants on the well-established, one-dimensional Visual Analog Scale (VAS-P). The VAS-P requires participants to mark a point on a 10-cm line, with 0 representing no pain and 10 representing the worst possible pain. Numerous studies have shown the VAS-P to be both reliable and valid [[22], [23], [24]].

### 2.8 Visual Analog Scale for anxiety

Anxiety levels were assessed using a standardized measurement tool known as the Visual Analog Scale for Anxiety (VAS-A). The scale is a straightforward 100-mm horizontal line ranging from “no anxiety” to “most anxiety”. The validity and reliability of the VAS-A have been substantiated through its demonstrated correlation with the Spielberger State-Trait Anxiety Inventory (STAI-Y1 and STAI-Y2) [25,26].

### 2.9 Intervention

The damask rose essential oil used (100 % pure, production license RD-PHL-COA-118) was obtained from Barijessence Pharmaceutical Company, Kashan, Iran. Considering the concentrated nature of undiluted essential oils, a 40 % dilution was achieved using Polyethylene Glycol 600 (PEG 600) as a neutral diluent. The non-therapeutic, odorless, oily substance PEG 600 was obtained from Barijessence Pharmaceutical Company (production license: RD-PHL-COA-118). The intervention group's treatment regimen comprised both rhythmic respiration techniques and aromatherapy. One-on-one bedside instruction was given on rhythmic breathing exercises; patients practiced slow, deep, rhythmic breaths following a 1:3:3 ratio for inhalation, breath-holding, and exhalation. The patient received aromatherapy via diluted Damask rose essential oil (40 %) on gauze pads attached to their collar. This procedure was repeated hourly for 3 h total. The control group received standard nursing care plus a placebo of carrier oil (PEG 600) on gauze, mimicking the intervention group's application method. Using the VAS, pain intensity and anxiety were measured at baseline, and 1-, 2-, and 3 h post-intervention.

### 2.10 Data analysis

The data analysis was performed with IBM SPSS Statistics version 22.0. Data normality was assessed by examining skewness and kurtosis. Normality was presumed; both values were in the −2 to +2 interval. Complete data resulted from the researcher's direct data collection. Categorical variables were compared using either the chi-squared test or Fisher's exact test, while continuous variables were analyzed using an independent samples *t*-test to assess demographic differences between groups. Using a repeated-measures analysis of variance (ANOVA), the investigators were able to analyze and determine the changes in pain intensity and anxiety levels across the various time points in the study. p < .05 was significant.

### 2.11 Ethical Considerations

The study was approved by the Ethics Committee of Zanjan University of Medical Sciences (Ethics Code: IR.ZUMS.REC.1403.036) and registered in the Iranian Registry of Clinical Trials (IRCT: IR.ZUMS.REC.1403.036). Written informed consent was obtained from all participants, and confidentiality of patient information was ensured.

## 3 Result

The final analysis included 56 patients, with 28 participants in each group (Fig. 1). There were no statistically significant differences between the intervention and control groups in terms of demographic or clinical characteristics at baseline (Table 1, Table 2), confirming group comparability before the intervention.

**Table 1:** Demographic characteristics of participants in the intervention and control groups.

Variables	Categories	Intervention *(n = 28)* n (%)	Control *(n = 28)* n (%)	*p*
Gender	Male	14 (50.0)	16 (57.1)	.592^a^
	Female	14 (50.0)	12 (42.9)	
Marital status	Single	5 (17.9)	6 (21.4)	.737^a^
	Married	23 (82.1)	22 (78.6)	
Education level	Illiterate	16 (57.1)	12 (42.9)	.332^b^
	Literacy in reading and writing	9 (32.1)	10 (35.7)	
	Primary school and over	1 (3.6)	5 (17.9)	
	Junior college and over	2 (7.1)	1 (3.6)	
Age	Mean (SD)	58.39 (8.33)	57.32 (6.93)	.341^c^

**Note.**^a^Chi-square test; ^b^Fisher's exact test; ^c^Independent *t*-test.

Percentages are based on the total number in each group.

**Table 2:** Clinical characteristics of participants in the intervention and control groups.

Variables	Categories	Intervention (n = 28) n (%)	Control (n = 28) n (%)	*p*
Underlying diseases	Yes	7 (25.0)	7 (25.0)	.621^a^
	No	21 (75.0)	21 (75.0)	
Sedative administration	None	11 (39.3)	20 (71.4)	.054^b^
	One dose	15 (53.6)	7 (25.0)	
	Two doses	2 (7.1)	1 (3.6)	
Type of sedative	Pethidine	8 (28.6)	3 (10.7)	.188^b^
	Morphine	5 (17.9)	4 (14.3)	
Cardiovascular drugs	Beta-blockers	21 (75.0)	24 (85.7)	.309^a^
	Calcium-channel blockers	4 (14.3)	4 (14.3)	1.000^b^
	Angiotensin receptor blockers	4 (14.3)	6 (21.4)	.488^b^
	Nitrates	3 (10.7)	4 (14.3)	.683^b^
Metabolic/Other drugs	Anti-lipid drugs	13 (46.4)	10 (35.7)	.417^a^
	Diabetes medications	6 (21.4)	12 (42.9)	.086^a^
	Benzodiazepines	7 (25.0)	3 (10.7)	.162^b^
	NSAIDs	5 (17.9)	0 (.0)	.053^b^
Lifestyle-related risk factors	Smoking	14 (50.0)	8 (28.6)	.101^a^
Baseline scores	Pain, Mean (SD)	7.50 (1.50)	6.71 (1.63)	.603^c^
	Anxiety, Mean (SD)	75.0 (17.74)	67.14 (17.18)	.481^c^

**Note.**^a^Chi-square test; ^b^Fisher's exact test; ^c^Independent *t*-test.

SD = Standard deviation. All percentages are based on the total number in each group.

Drug classes are categorized according to the original dataset.

**Fig. 2:**
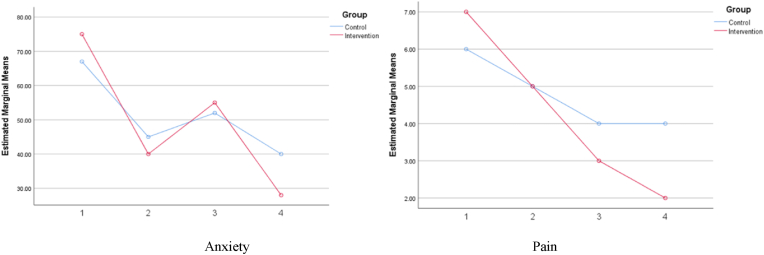
Comparison of Pain and anxiety of intervention, and control groups.

At baseline, the mean anxiety score was 75.00 (17.74) in the intervention group and 67.14 (17.18) in the control group. Repeated-measures ANOVA indicated a significant reduction in anxiety over time in both groups (P < .001). However, the intervention group showed a more pronounced improvement over time compared with the control group, confirming a significant group × time interaction (P < .001). Overall, anxiety scores decreased by 61.4 % in the intervention group versus 40.2 % in the control group over the 3-h follow-up period (Table 3 and Fig. 2).

**Table 3:** Comparison of anxiety scores between groups across four time points.

Time point	Intervention Mean (SD)	Control Mean (SD)	Interaction *P*	Within-group *P*
Baseline	75.00 (17.74)	67.47 (17.18)	<.001	<.001
1 h	55.35 (15.74)	52.50 (16.91)		
2 h	40.71 (18.24)	45.71 (19.51)		
3 h	28.92 (16.17)	40.35 (19.33)		

**Note.** Repeated–measures ANOVA was used to assess within–group and group × time interaction effects.

Similar findings were observed for pain intensity. At baseline, pain intensity was 7.50 (1.50) in the intervention group and 6.71 (1.63) in the control group. Pain significantly declined within both groups over time (P < .001), and the pattern of change differed significantly between the two groups (P < .001, interaction effect), favoring the intervention. Pain scores decreased by 61.5 % in the intervention group compared with 36.2 % in the control group (Table 4 and Fig. 2).

**Table 4:** Comparison of pain scores between groups across four time points.

Time point	Intervention Mean (SD)	Control Mean (SD)	Interaction *P*	Within-group *P*
Baseline	7.50 (1.50)	6.71 (1.62)	<.001	<.001
1 h	5.42 (1.59)	7.14 (10.46)		
2 h	3.71 (1.48)	4.60 (1.49)		
3 h	2.89 (1.31)	4.28 (1.58)		

**Note.** Data analyzed using repeated–measures ANOVA.

The detailed temporal changes in anxiety and pain scores across the four measurement time points are presented in Table 3, Table 4

## 4 Discussion

The findings of this randomized clinical trial showed that the combined application of rhythmic breathing and rose essential oil aromatherapy resulted in significant improvements in both anxiety and pain intensity among patients with unstable angina. This study provides new evidence supporting the use of simple, fast, and non-pharmacological interventions in acute cardiac care, especially in coronary care units where anxiety and pain are common and can worsen myocardial ischemia.

Anxiety plays a major physiological role in exacerbating cardiac symptoms by increasing sympathetic activity, elevating heart rate, and raising myocardial oxygen demand. Therefore, reducing anxiety is a critical therapeutic priority in acute coronary syndrome (ACS) [27]. In our study, anxiety scores decreased by 61.4 % in the intervention group compared with 40.2 % in the control group within just 3 h, highlighting a rapid clinical effect. These results are consistent with previous studies demonstrating that rhythmic breathing enhances vagal tone, improves respiratory efficiency, and decreases stress-induced catecholamine release in ACS patients [28,29].

Aromatherapy has been proposed as an adjunct therapy for anxiety management in cardiovascular patients by influencing limbic system activity and reducing sympathetic arousal. Several studies have confirmed that rose essential oil has anxiolytic effects in ACS and post-angioplasty patients [[30], [31], [32]]. Our findings expand this evidence by revealing that **integrating** breathing and aromatherapy can generate a **synergistic** therapeutic impact. Rhythmic breathing facilitates deeper inhalation and enhances the physiological uptake of aromatic molecules through the olfactory pathway, potentially strengthening their calming effect.

Pain relief is another crucial outcome. Chest pain contributes directly to clinical instability and emotional fear in unstable angina. In our study, pain was reduced by 61.5 % in the intervention group versus 36.2 % in the control group, supporting the superiority of the combined technique. These findings are consistent with previous research indicating that slow-breathing exercises can reduce perceived pain and improve autonomic regulation in cardiac patients [33,34]. Furthermore, the analgesic effect of rose aromatherapy, potentially mediated through serotonergic and endorphin pathways, has been demonstrated in clinical settings, including significant pain relief in patients with myocardial infarction [[35], [36], [37]].

From a nursing perspective, this intervention is highly practical because it does not require specialized equipment, pharmacological preparation, or additional personnel. It can be easily implemented at the bedside by nurses, particularly when medication effects may be delayed or contraindicated. Furthermore, it aligns with the growing emphasis on patient-centered and holistic care in cardiovascular nursing.

The synergistic effects observed in this study can be explained by physiological mechanisms associated with both interventions. Rhythmic breathing enhances baroreflex sensitivity and increases parasympathetic activation, which leads to reduced heart rate, improved coronary perfusion, and lower myocardial oxygen demand. Meanwhile, rose essential oil exerts anxiolytic effects by stimulating olfactory receptors and influencing the limbic system, particularly the amygdala and hypothalamus, thereby reducing sympathetic dominance and stress-related catecholamine release. The combined intervention likely optimizes autonomic nervous system balance and evokes a relaxation response more effectively than either method alone. This mechanism may explain the rapid reduction of anxiety and associated ischemic pain in the intervention group.

### 4.1 Strengths and Limitations

A major strength of this study is its randomized controlled design and repeated measurements, allowing robust evaluation of symptom progression over time. The simultaneous assessment of anxiety and pain adds to the clinical relevance of our findings.

However, the short monitoring period may not reflect longer-term outcomes. Additionally, the sample was limited to two hospitals in a single geographic region, which may affect generalizability. Blinding of participants was not feasible due to the nature of the intervention, which may introduce placebo effects. Future research should incorporate longer follow-up durations, physiological measures such as HRV, and comparisons between different essential oils.

## 5 Conclusion

Overall, the combination of rhythmic breathing and rose aromatherapy is an effective, safe, and low-cost complementary therapy for reducing anxiety and pain intensity in patients with unstable angina. These results support the integration of such interventions into routine cardiac nursing practice to enhance patient comfort and prevent anxiety-driven ischemic complications. Larger multi-center trials are recommended to confirm these benefits and facilitate implementation in broader cardiac care settings.

## CRediT authorship contribution statement

**Sahar Nouri:** Writing – original draft, Visualization, Resources, Investigation, Funding acquisition, Data curation, Conceptualization. **Nasrin Hanifi:** Writing – review & editing, Validation, Supervision, Software, Methodology, Formal analysis, Data curation, Conceptualization. **Farhad Ramezani-Badr:** Writing – review & editing, Supervision, Methodology, Formal analysis, Conceptualization.

## Declaration of competing interest

The authors declare the following financial interests/personal relationships which may be considered as potential competing interests:Travel expensesEquipment supply.

## Data Availability

Data will be made available on request.
